# Ultra-low dose superparamagnetic iron oxide nanoparticle injection for sentinel lymph node detection in breast cancer: prospective cohort study

**DOI:** 10.1093/bjs/znaf129

**Published:** 2025-07-23

**Authors:** Lovisa Sundh, Marya Alzoubi, Nizar Abu-Oddos, Sarah Båtelsson, Per Nyman, Andreas Karakatsanis, Staffan Eriksson, Mookaiah Ravichandran, Kian Chin, Roger Olofsson Bagge, Nushin Mirzaei, Fredrik Wärnberg

**Affiliations:** Department of Surgery, Region Västra Götaland, Sahlgrenska University Hospital, Gothenburg, Sweden; Department of Surgery, Institute of Clinical Sciences, Sahlgrenska Academy, University of Gothenburg, Gothenburg, Sweden; Department of Surgery, Institute of Clinical Sciences, Sahlgrenska Academy, University of Gothenburg, Gothenburg, Sweden; Department of Surgery, Region Västra Götaland, Sahlgrenska University Hospital, Gothenburg, Sweden; Section for Breast Surgery, Department of Surgery, Västmanland County Hospital, Västerås, Sweden; Department of Surgery, Skaraborg Hospital, Lidköping, Sweden; Section for Breast Surgery, Department of Surgical Sciences, Uppsala University Hospital, Uppsala, Sweden; Department of Surgical Sciences, Uppsala University, Uppsala, Sweden; Section for Breast Surgery, Department of Surgery, Västmanland County Hospital, Västerås, Sweden; Section for Breast Surgery, Department of Surgical Sciences, Uppsala University Hospital, Uppsala, Sweden; Department of Surgery, Skaraborg Hospital, Lidköping, Sweden; Department of Surgery, Region Västra Götaland, Sahlgrenska University Hospital, Gothenburg, Sweden; Department of Surgery, Institute of Clinical Sciences, Sahlgrenska Academy, University of Gothenburg, Gothenburg, Sweden; Department of Surgery, Region Västra Götaland, Sahlgrenska University Hospital, Gothenburg, Sweden; Department of Surgery, Institute of Clinical Sciences, Sahlgrenska Academy, University of Gothenburg, Gothenburg, Sweden; Wallenberg Centre for Molecular and Translational Medicine, University of Gothenburg, Gothenburg, Sweden; Department of Surgery, Region Västra Götaland, Sahlgrenska University Hospital, Gothenburg, Sweden; Department of Surgery, Institute of Clinical Sciences, Sahlgrenska Academy, University of Gothenburg, Gothenburg, Sweden; Department of Surgery, Region Västra Götaland, Sahlgrenska University Hospital, Gothenburg, Sweden; Department of Surgery, Institute of Clinical Sciences, Sahlgrenska Academy, University of Gothenburg, Gothenburg, Sweden

## Abstract

**Background:**

Sentinel lymph node (SLN) staging is essential in breast cancer. Superparamagnetic iron oxide nanoparticles (SPIO) is a tracer where the optimal injection technique is yet not defined. The aim was to evaluate SLN detection using 0.1 ml SPIO intradermally compared to technetium-99 m (Tc^99^) ± blue dye.

**Method:**

Patients planned for breast surgery and SLN biopsy received 0.1 ml SPIO intradermally at the areolar border or over the tumour. Tc^99^ ± blue dye was administered per clinical routine. Magnetic, radioactive, or blue nodes were removed and analysed separately. SLN detection and numbers, concordance, and skin discoloration were analysed.

**Results:**

A total of 216 patients were included at five hospitals. Median age was 63 years, tumour size 15.9 mm, and 91.7% underwent breast conservation. SPIO was injected a median of 12 days before surgery. SLN detection was 211/216 (97.7%; 95% c.i.: 94.7 to 99.2) and 215/216 (99.5%; 95% c.i.: 98.6 to 100.0) for SPIO and Tc^99^ ± blue dye (*P* = 0.111) respectively. In total, 403 SLNs were removed; 341 detected by SPIO and 349 by Tc^99^ ± blue dye. The median number of SLNs was 1 (iqr: 1–2) for both tracer methods. Among 46 SLN-positive patients, 42 were correctly staged with both tracers, two with SPIO only and two with Tc^99^ ± blue dye only. Skin discoloration was evaluated in 107 patients. The median discoloured area was 0 cm^2^ (iqr: 0–0.7) among 49 patients with the injection site surgically removed and 1.3 cm^2^ (iqr: 0.6–2.8) among 58 without removal.

**Conclusion:**

An ultra-low dose of 0.1 ml intradermal injection of SPIO was non-inferior to Tc^99^ ± blue dye for SLN detection. Skin discoloration was limited and further reduced by removal during surgery.

## Teaser

In a prospective cohort study we compared 0.1 ml Magtrace with standard sentinel lymph node (SLN) tracers. The ultra-low dose of Magtrace was injected intracutaneously and was found to be non-inferior to standard. The SLN detection was 97.7% and 99.5% respectively.

## Introduction

Sentinel lymph node (SLN) detection in breast cancer patients using superparamagnetic iron oxide nanoparticles (SPIO) has been shown to be as effective as former gold standard using technetium^99m^ (Tc^99^), with or without blue dye (BD)^1^. SPIO offers several logistic advantages; the tracer remains detectable for a long period after administration and does not require the nuclear medicine facilities that are needed for Tc^99^ detection, and compared to BD no severe allergic reactions have been reported^1–2^.

When first introduced as an SLN tracer, SPIO was injected behind the areola and the recommended volume was 5 ml (2 ml SPIO diluted in 3 ml saline)^3^. SPIO can cause skin discoloration^4^ and postoperative MRI artefacts^5^ in the breast parenchyma. Subsequently, based on clinical studies, the recommended injection volume has been reduced, and the injection technique has been refined^6^. Today, 1 ml SPIO injected deeper close to the tumour is routine at many sites. To further reduce discoloration and artefacts, an ultra-low dose (0.1 ml) of SPIO was injected intradermally in a pilot study including 50 patients with 100% SLN detection^7^.

The primary aim of this study was to confirm non-inferior SLN detection using an ultra-low dose of 0.1 ml SPIO injected intradermally in patients with breast cancer compared to clinical routine with Tc^99^ ± BD. The secondary aim was to evaluate short-term skin discoloration.

## Method

### Study population and design

In this multicentre prospective cohort study conducted in five Swedish hospitals (hospitals A–E) between March 2023 and May 2024, clinically node negative patients (cN0) scheduled for breast surgery and an SLN biopsy (SLNB) received both 0.1 ml SPIO and Tc^99^ ± BD, following written informed consent. Blue dye was used according to clinical practice at the different hospitals and two of the hospitals (B and C) were considered as one unit with similar routines. Exclusion criteria were age under 18 years, pregnancy or breast-feeding, hypersensitivity to any of the SLN tracers, or iron overload disease. The study was registered at clinicaltrials.gov (NCT06169072) following approval from the Swedish Ethical Review Authority (Dnr: 2021-02726 and 2022-05239-02) and the Swedish Medical Product Agency (Dnr 5.1-2022-95550).

### SPIO, technetium^99^, and blue dye tracer injections

An intradermal injection of 0.1 ml SPIO (Magtrace®, Endomagnetics Ltd, Cambridge, UK) was administered at the border of the areola or over the tumour site up to 30 days prior to surgery. Tc^99^ and BD (Patent Blue V®, Guerbet, France) were injected per clinical routine. Tc^99^ at the intra-/subdermal border of the areola or over the tumour site, either the day before (60–120 MBq) or on the day of surgery (40 MBq), and BD (0.1–2.0 ml sub-/intracutaneously at the areolar border) after the onset of anaesthesia.

### Surgery

The approach was to first identify SLNs with the magnetic technique, that is SPIO and the SentiMag-probe (Endomagnetics Ltd, Cambridge, UK) and thereafter with Tc^99^ ± BD and a gamma probe (Neo2000®/Neoprobe 2100, Neoprobe, USA or Gamma Finder®, World of Medicine, Germany). Magnetic and radioactive counts were registered for every SLN, both still in place in the axilla and after removal (*in vivo* and *ex vivo*). Lymph nodes containing more than 10% activity compared to the lymph node with the maximum activity, for both SPIO and Tc^99^, as well as BD-stained lymph nodes were considered SLNs. Additionally, only nodes reaching a signal of at least 20 counts were considered SLNs for that tracer method. Nodes without magnetic signal radioactive signal or blue stained but with a suspicion of being metastatic were also removed but were not classified as SLNs and were not included in the analyses of SLN detection per tracer. Each SLN was sent separately for routine histopathology.

### Skin discoloration

Surgical removal of the injected skin area was optional and was recorded. The remaining area (cm^2^) of skin discoloration was registered at the first follow-up visit after 4–5 weeks. Patient-reported experience of the discoloration, according to the modified questionnaire Skin Discoloration Impact Evaluation Questionnaire (SDIEQ)^8^ and skin discoloration over time, will be followed up to three years.

### Statistical analysis

The overall aim was to evaluate whether an ultra-low dose of 0.1 ml SPIO injected intradermally was non-inferior to Tc99 ± BD for SLN detection. The SLN detection ratio was estimated to be 98% for both SPIO and Tc^99^ ± BD based on our previous pilot study where all patients had a successful SLNB^7^. Acceptable non-inferiority margins for SLN detection SLN detection was set at 4%. If there is truly no difference between the two methods, then 193 patients would be required in a study with 80% power and a one-sided 97.5% confidence interval to exclude a difference in favour of the Tc^99^ ± BD of more than 4%. Allowing for dropouts of approximately 10%, up to 220 patients were planned to be recruited.

Categorical variables were summarized by numbers and percentages with 95% confidence intervals, and continuous variables by medians and ranges. Continuous variables were compared with the Wilcoxon test for paired outcomes. For SLN detection, McNemar’s test was used to compare SPIO *versus* Tc^99^ ± BD. Concordance was determined as the number of SLNs detected by both Tc^99^ ± BD and SPIO divided by the number of all SLNs detected by Tc^99^ ± BD. Reverse concordance was defined as the number of SLNs detected by both SPIO and Tc^99^ ± BD, divided by the number of all SLNs detected by SPIO. *P* < 0.05 was considered statistically significant.

## Results

In total, 217 patients were enrolled in the study. One patient did not meet the inclusion criteria and was excluded (*Fig. S1*), leading to a final study cohort of 216 patients. Patient and primary tumour characteristics are presented in *Table 1*. Two patients had previously undergone breast surgery and 91.7% (198/216) underwent breast-conserving surgery as the final procedure. SPIO was injected a median of 12 days before surgery (range: 0–36 days). In one patient surgery was postponed and performed two weeks later.

**Table 1:** Patient and tumour characteristics among 216 patients undergoing sentinel lymph node biopsy using an ultra-low dose of SPIO

Age, years, median (range)	63 (31–87)
BMI, kg/m^2^, median (range)	25.1 (15.9–54.7)
BCS/Mx, number	198/18
Tumour size, mm, median (range)	15.9 (2.0–75.0)
**Tumour grade, number (%)**	
Grade1	45 (20.8%)
Grade 2	134 (62.0%)
Grade 3	33 (15.2%)
Missing	4 (1.8%)
**Tumour type**	
Ductal	158 (73.0%)
Lobular	25 (11.6%)
Other	26 (12.0%)
*In situ*	2 (0.9%)
Missing	2 (0.9%)
ER+ ≥10%	206 (95.4%)
PR+ ≥10%	175 (81.1%)
HER2+*, *n* = 210	11 (5.2%)
**Ki-67**	
≤5%	37 (17.1%)
6–29%	142 (65.7%)
≥30%	37 (17.1%)

^*^HER2-positive, immunohistochemistry 3+ or amplified. SPIO = supraparamagnetic nanoparticles of iron oxide, BCS = breast-conserving surgery, Mx = mastectomy.

Detection of at least one SLN using SPIO was successful in 211 of 216 patients (97.7%; 95% c.i.: 94.7 to 99.0), whereas Tc^99^ ± BD detected at least one SLN in 215 of 216 patients (99.5%; 95% c.i.: 97.4 to 100.0). The difference between the methods was 1.8% (95% c.i.: −0.8 to 4.5, *P* = 0.111), confirming that the primary endpoint of non-inferiority was met (*Table 2*). In 210 patients, both tracers (SPIO and Tc^99^ ± BD) were successful, resulting in a concordance of 100% (95% c.i.: 98.3 to 100.0) and a reverse concordance of 99.5% (95% c.i.: 94.7 to 99.7) (*Table 2*). Blue dye was used in 59 patients and at least one blue node was detected in 40 patients (67.8%). Two patients had a blue node without a radioactive signal. One patient had no SLNs identified using either SPIO, Tc^99^, or BD. In this patient a sampling was performed with only one non-SLN removed.

**Table 2:** SLN detection among 216 patients injected with both 0.1 ml SPIO intradermally and Tc^99^, with or without blue dye

	SPIO 0.1 mL	Tc^99^ ± BD
Successful SLN detection, % (95% c.i.), *n* = 216	97.7% (94.7–99.0)	99.5% (97.4–100.0)
Number of removed SLNs per patient, median (iqr)	1 (1–2)	1 (1–2)
Concordance*/reverse concordance†, % (95% c.i.)		
Per patient, *n* = 216	100% (98.3–100.0)/99.5% (94.7–99.7)	
Per SLN, *n* = 403	82.2% (78.8–87.0)/84.2% (79.8–87.9)	
Per SLN-positive patient, *n* = 46	96% (84–99)/96% (84–99)	
Per positive SLN, *n* = 61	86% (74–94)/83% (71–92)	

^*^Concordance is all patients (or lymph nodes) detected with both Tc^99^ ± BD and SPIO divided by all patients (or lymph nodes) detected with Tc^99^ ± BD. †Reverse concordance is all patients (or lymph nodes) detected with both Tc^99^ ± BD and SPIO divided by all patients (or lymph nodes) detected with SPIO. Abbreviations: BD = blue dye; SLN = sentinel lymph node; SPIO = superparamagnetic iron oxide nanoparticles; Tc^99^ = technetium^99m^.

For the different hospitals, the SLN detection ratio for SPIO *versus* Tc^99^ ± BD were: Hospital A, 98.7% (155/157) *versus* 100% (157/157), *P* = 0.480; Hospitals B + C, 94% (33/35) *versus* 97% (34/35), *P* = 1.0; and Hospital D+E, 96% (23/24) *versus* 100% (24/24), *P* = 1.0, respectively.

A total of 465 lymph nodes were removed with a median of 2 nodes per patient (iqr: 2–2) and of those 465 lymph nodes, 403 were classified as SLNs by any of the tracers. SPIO detected 341 nodes with a median of 1 (iqr: 1–2) SLN per patient and 349 nodes were detected with Tc^99^ ± BD with a median of 1 (iqr: 1–2). Both tracer methods detected 287 SLNs, giving a concordance per node of 82.2% (95% c.i.: 78.8 to 87.0) and a reverse concordance of 84.2% (95% c.i.: 79.8 to 87.9) (*Table 2*).

The preoperative transcutaneous axillary signal was in median 107 (iqr: 57–170) for SPIO and 165 (iqr: 60–500) for Tc^99^. For the highest signalling SPIO-SLN the median *in vivo* signal was 359 (iqr: 100–1300) and the median *ex vivo* signal was 1500 (iqr: 670–2505). The SLN was registered as brown in 67.0% (118/176) of the cases. The median *in vivo* Tc^99^ signal in the highest signalling Tc^99^-SLN was 388 (iqr: 256–1200) and *ex vivo* 844 (iqr: 365–2740). In cases where BD was used, the Tc^99^-SLNs were registered as blue in 59.3% (35/59) of the patients.

Forty-six patients were classified as SLN-positive with either SPIO or Tc^99^ ± BD with at least one macro- or micrometastatic SLN. Another five patients had exclusively non-SLN metastases. Among the 46 patients with SLN metastases, 42 had at least one metastatic SLN detected with both tracer methods, two only with SPIO and two only with Tc^99^ ± BD, giving both a concordance and reverse concordance per node-positive patient of 96% (95% c.i. 84 to 99). Of the total 71 metastatic lymph nodes, ten were non-SLNs. Both tracer methods detected 45 of the metastatic nodes, seven with SPIO only, and nine with Tc^99^ ± BD only, giving a concordance of 86% (95% c.i.: 74 to 94) and a reverse concordance of 83% (95% c.i.: 71 to 92) per metastatic node (*Table 2*).

**Fig. 1:**
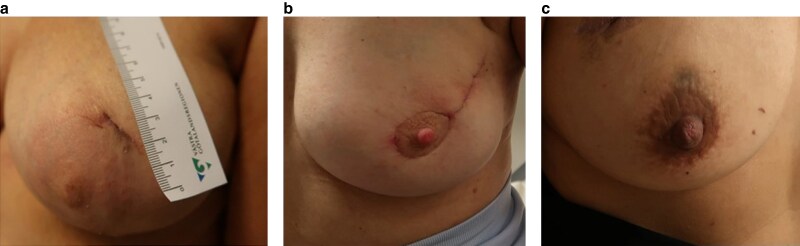
a–c Skin discoloration six weeks after surgery in patients injected with 0.1 ml SPIO (Magtrace®, Endomagnetics Ltd, UK) intradermally, and who had the discoloration surgically removed during surgery (**a**,**b**). The third patient also had 0.1 ml SPIO injected intradermally, but the discoloration was not removed during surgery (**c**).

All patients had some skin discoloration after their SPIO injection. In 83 of 175 (47.4%) undergoing breast-conserving surgery the skin discoloration, or part of it, was removed during surgery. Another 23 (13.1%) patients undergoing breast-conserving surgery had missing data regarding skin removal. Data on both skin removal and skin discoloration at the first postoperative visit was available for 107 (61.1%) patients. The remaining discoloured skin area for those 49 (45.8%) patients where the discoloured skin or part of it was removed was in median 0 cm^2^ (iqr: 0–0.7) and for those 58 (54.2%) where the skin discoloration was not removed, the median area was 1.3 cm^2^ (iqr: 0.6–2.8; *P* = 0.092) (*Fig. 1a–c*). No severe adverse effects of either SPIO or Tc^99^ ± BD were reported.

## Discussion

This prospective multicentre cohort study demonstrates that SLN detection using an ultra-low dose of 0.1 ml SPIO injected intradermally was non-inferior to Tc^99^ ± BD, with detection ratios of 97.7% and 99.5% respectively.

The median numbers of SLNs detected with 0.1 ml SPIO injected up to 30 days before surgery (median 12 days), compared to Tc^99^ ± BD injected according to routine practice, were similar. The 0.1 ml SPIO dose did not result in fewer nodes being removed compared with Tc^99^ ± BD. Additionally, the longer time span between injection of SPIO and surgery did not result in more SLNs being removed. The median number of SLNs was one (1) for both tracer methods but including the non-SLNs resulted in removal of a median of two nodes (mean 2.2) per SLNB procedure. It is known that removal of more SLNs reduces the false negative rate^9–10^; however, in more than half of the patients in the present study only one SLN was found using the 10% cutoff rule^9^ even if all three tracers (SPIO, Tc^99^, and BD) were used. In this study it was not possible to calculate the false negative rate as there were no axillary lymph node clearances after the SLNB.

In a pooled patient-level analysis by Christenhusz *et al*.^11^ including 908 patients from eight studies in Sweden and the Netherlands using SPIO and Tc^99^, the median and mean number of SLNs removed were 2 and 1.89 respectively, but non-SLNs were not reported. In a meta-analysis of seven early studies comparing SPIO with standard radioactive tracer^12^, the mean number of removed SLNs were 2.06 (2300/1118). In a randomized study by Man *et al*.^13^ the mean number of removed SLN was 3.3 in the study group receiving SPIO and 2.8 in the control group receiving Tc^99^. It can be difficult to compare the results from different studies depending on the setting. For example, in the study by Man *et al*., 2.0 ml of SPIO tracer was injected subareolarly, and in the study by Christenhusz *et al*. different doses, injection sites, and time frames were used.

The concordance and reverse concordance in the present study were high per patient and lower per node, similar to the results from the study by Man *et al*.^13^. Furthermore, in the study by Christenhusz *et al*.^11^ concordance and reverse concordance between SPIO and Tc^99^ (without BD) for SLN detection per patient were both 97.2%, which is somewhat lower than in this study. For SLN detection per node in the Christenhusz study, concordance and reverse concordance were 91.4% and 86.6% compared to 82.2% and 84.2% in the present study, indicating that the proportion of nodes detected with both tracers was lower. However, the majority of SLNs were detected with both tracer methods.

Of all metastatic lymph nodes removed in this study, 72% were detected by SPIO and 75% by Tc^99^ ± BD. Both methods correctly staged 86% of the patients as N+ with a concordance and reverse concordance per N+ patient of 96%. In the randomized study by Man *et al*.^13^ the concordance and reverse concordance per N+ patient was 97.3% in the study group receiving both SPIO and Tc^99^. The concordance and reverse concordance per malignant SLN were 96.8% and 92.3% respectively compared to 86% and 83% in the present study. However, only the *ex vivo* signal for Tc^99^ was used in the study group by Man *et al*. This could explain the higher concordance and reverse concordance in their study group. Of 240 SLNs containing metastases or isolated tumour cells in the study of Christenhusz *et al*.^11^, 68.3% had recorded counts for both SPIO and Tc^99^ and the SLN-based concordance and reverse concordance for metastatic SLNs were 98.6%, and 88.3% respectively.

Several dose/volume-reducing studies have been conducted since the introduction of SPIO. In this study a 50-times lower volume compared to the first clinical studies was used^2,12^. One reason for reducing the volume has been to reduce skin discoloration even though it seems that women do not consider the discoloration a major problem^14^. In a meta-analysis by Pantiora *et al*., the injected volume did not affect the SLN detection^15^. The analysis did not, however, include data for 0.1 ml SPIO and to the authors’ knowledge there are very few patients reported where 0.1 ml SPIO was used as an intratumoural injection^16^. In two pilot studies conducted in breast cancer and melanoma patients a 0.1 ml intradermal SPIO injection with high SLN detection was used^7,17^. Injecting SPIO into the skin, however, inevitably causes some discoloration, although it appears possible to reduce postoperative discoloration by removing the affected skin during surgery. If a peri-areolar incision is planned and a peri-areolar injection is used, this is easily done (*Fig. 1a,b*). In patients undergoing mastectomy, the injection site could be planned to be included in the removed skin. In this study, 0.1 ml SPIO was compared with Tc^99^ with or without blue dye. The blue dye skin discoloration was not registered. In an earlier study it was shown that if used, the blue dye causes skin discoloration to the same extent as 1–2 ml SPIO and the discoloration lasts for a similar length of time^14^.

Another intention of injecting SPIO intradermally in a very low dose is to limit MRI artefacts. Smaller volumes injected close to the tumour will often be more or less entirely removed with the surgical specimen, reducing MRI artefacts^18^. In a follow-up of the authors’ pilot study, the effect on the interpretation of postoperative breast MRIs of the intradermal superficial injection is evaluated and results are awaited.

Many tumours in this study were non-palpable and underwent preoperative localization. There was no specific protocol for tumour localization, both guidewires and magnetic clips (Magseed®, Endomagnetics Ltd, Cambridge, UK) were used. The clips were inserted by the radiologist in the ventral part of the tumour as part of clinical routine and SPIO was injected at least 2–3 cm away from the tumour/clip area by the surgeon or a research nurse. Further, the 0.1 ml SPIO dose would decrease the cost of the tracer per patient, although 0.1 ml is currently off label.

One limitation of this study is that blue dye was not used in a prespecified way. There was a resistance among many surgeons to use the BD as it is not used routinely today in all breast units, and a pragmatic stance was chosen to facilitate patient inclusion. Moreover, even if the SLN detection using BD in addition to Tc^99^ was 10.4% higher in the ALMANAC validation phase^19^ and 5.1% higher in the NSABP B-32 study^9^, the overall SLN detection was 96.1% and 97.0% in these studies respectively. This was lower than in the present study, even for each tracer method separately, that is SPIO only, Tc^99^ only, and Tc^99^ ± BD. In the ALMANAC study 2 ml of blue dye (Patent Blue V) diluted in 5 ml of saline and in NSABP B-32 5 ml of undiluted isosulfan blue was injected peritumourally. The authors cannot rule out that using the different tracers in parallel could have generated higher SLN detection as one method could facilitate the identification of an SLN with another tracer. The protocol, however, stated that the magnetic method should be used first and a cutoff of a signal of at least 20 for both radioactivity and magnetic signals was set.

In each patient, a comparison of 0.1 ml SPIO to the routine tracer method that otherwise would have been used in that patient was done. Three patients would have had an unsuccessful SLNB if only Tc^99^ had been used. Two of those were detected with BD but also with SPIO, and the third was not detected with either BD or SPIO. No patient had a successful SLNB based on blue dye only. In the Christenhusz *et al*. study^11^, only 4 of 567 (0.7%) patients had a blue SLN without a radioactive signal. Among 1110 SLNs, 40 were blue but with no radioactivity (3.6%). This can be interpretated as the use of blue dye not affecting the present results in a clinically significant way. A strength of the study is the generalizability of the results. Five hospitals were involved, and the inclusion criteria covered almost all patients undergoing an SLNB.

SLN detection with an ultra-low 0.1 ml intradermal SPIO injection in women with breast cancer was thus non-inferior to standard radioactive tracer with or without blue dye. Skin discoloration was limited and can be even further reduced by removing the stained area at surgery.

## Supplementary Material

znaf129_Supplementary_Data

## Data Availability

Data can be made available upon reasonable request following contact with the corresponding author.

## Previous communication

Preliminary results from this study have been presented at the Swedish Surgical Week, August 2024, at the Leura International Breast Cancer Conference, October 2024, Australia, and at the 19th St. Gallen Breast Cancer Conference March 2025, Vienna, Austria.
