# Supplementary material for: Ultra-low dose superparamagnetic iron oxide nanoparticle injection for sentinel lymph node detection in breast cancer: prospective cohort study
Source: Br J Surg. 2025 Jul 23;112(7):znaf129. doi: 10.1093/bjs/znaf129 (PMC12284759; doi:10.1093/bjs/znaf129)

**Intradermal injection of an ultra-low dose of superparamagnetic iron oxide nanoparticles for sentinel lymph node detection in breast cancer patients: prospective cohort study**

*Lovisa Sundh ^1,2^, *Marya Alzoubi ^2^, Sarah Båtelsson ^3^, Per Nyman ^4^, Andreas Karakatsanis ^5,6^, Staffan Eriksson ^3,5^, Mookaiah Ravichandran ^4^, Kian Chin ^1,2^, Roger Olofsson Bagge ^1,2,7^, Nushin Mirzaei ^1,2^, Nizar Abu-Oddos ^1^, Fredrik Wärnberg ^1,2^

^1^ Department of Surgery, Sahlgrenska University Hospital, Gothenburg, Sweden

^2^ Sahlgrenska Centre for Cancer Research, Institute of Clinical Sciences, Sahlgrenska Academy at Gothenburg University, Gothenburg, Sweden

^3^ Section for Breast Surgery, Department of Surgery, Västmanland´s County Hospital, Västerås, Sweden

^4^ Department of Surgery, Skaraborg Hospital, Lidköping, Sweden

^5^ Section for Breast Surgery, Department of Surgical Sciences, Uppsala University Hospital, Uppsala, Sweden

^6^ Department of Surgical Sciences, Uppsala University, Uppsala, Sweden

^7^ Wallenberg Centre for Molecular and Translational Medicine, University of Gothenburg, Gothenburg, Sweden.

*Shared first authorship

**Corresponding author.**

Fredrik Wärnberg, Department of Surgery, Sahlgrenska University Hospital, S-413 45, Gothenburg, Sweden. e-mail: [fredrik.warnberg@vgregion.se](mailto:fredrik.warnberg@vgregion.se) ORCID 0000-0002-0130-7296

**Supplementary Materials - Index**

| **Supplementary Figures and Tables** |  |
| --- | --- |
| Figure 1, Flow chart | *page 1* |


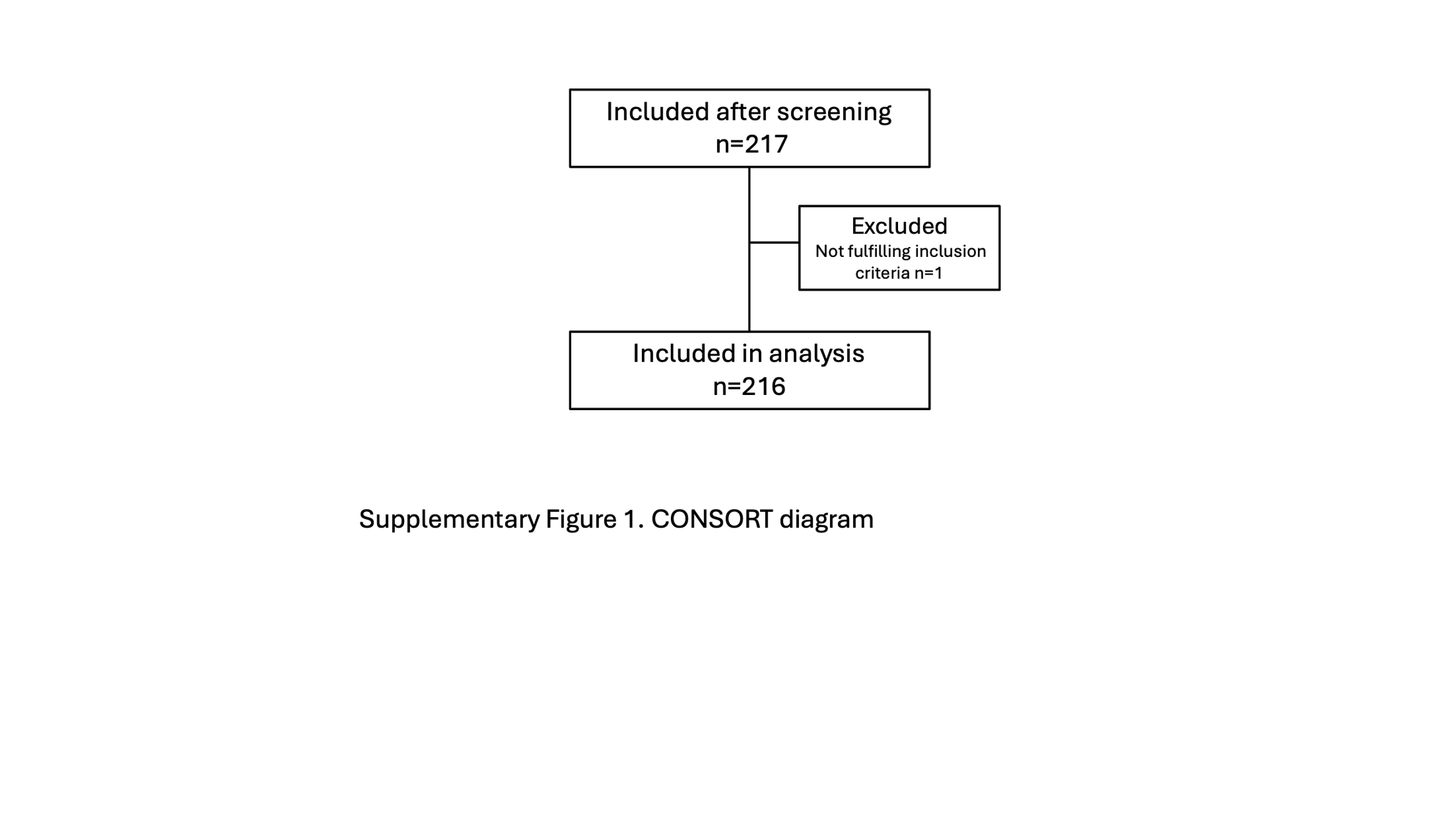

Supplement: znaf129_Supplementary_Data [file znaf129_supplementary_data.docx]
